# Autistic Children Exhibit Decreased Levels of Essential Fatty Acids in Red Blood Cells

**DOI:** 10.3390/ijms160510061

**Published:** 2015-05-04

**Authors:** Sarah A. Brigandi, Hong Shao, Steven Y. Qian, Yiping Shen, Bai-Lin Wu, Jing X. Kang

**Affiliations:** 1 Laboratory of Lipid Medicine and Technology, Department of Medicine, Massachusetts General Hospital and Harvard Medical School, Boston, MA 02129, USA; E-Mail: sarah.brigandi@gmail.com; 2 Genetics Diagnostic Lab, Department of Laboratory Medicine and Pathology, Children’s Hospital Boston and Harvard Medical School, Boston, MA 02114, USA; E-Mails: hongshao138@gmail.com (H.S.); yiping.shen@childrens.harvard.edu (Y.S.); bai-lin.wu@childrens.harvard.edu (B.-L.W.); 3 Department of Pharmaceutical Science, North Dakota State University, Fargo, ND 58108, USA; E-Mail: steven.qian@ndsu.edu

**Keywords:** polyunsaturated fatty acids, omega-6 and omega-3 fatty acids, prostaglandin E2, lipid metabolism, neuroinflammation, autism

## Abstract

Omega-6 (n-6) and omega-3 (n-3) polyunsaturated fatty acids (PUFA) are essential nutrients for brain development and function. However, whether or not the levels of these fatty acids are altered in individuals with autism remains debatable. In this study, we compared the fatty acid contents between 121 autistic patients and 110 non-autistic, non-developmentally delayed controls, aged 3–17. Analysis of the fatty acid composition of red blood cell (RBC) membrane phospholipids showed that the percentage of total PUFA was lower in autistic patients than in controls; levels of n-6 arachidonic acid (AA) and n-3 docosahexaenoic acid (DHA) were particularly decreased (*p* < 0.001). In addition, plasma levels of the pro-inflammatory AA metabolite prostaglandin E2 (PGE2) were higher in a subset of the autistic participants (*n =* 20) compared to controls. Our study demonstrates an alteration in the PUFA profile and increased production of a PUFA-derived metabolite in autistic patients, supporting the hypothesis that abnormal lipid metabolism is implicated in autism.

## 1. Introduction

Autism spectrum disorders (ASD) are a group of related neurological developmental disorders that affect approximately 45 to 110 individuals per 10,000 [1]. ASD includes Autism, Asperger Syndrome, and pervasive developmental disorder, not otherwise specified (PDD-NOS). Autism symptoms arise early in childhood and include varying degrees of communication and social interaction impairments, limited and repetitive behaviors, and often issues with motor coordination [2,3]. Autism has been classified as a heterogeneous disease that likely results from the complex interaction of multiple genes with environmental factors [4]. While some studies suggest that genetics play a role in the majority of autism cases [4], only 10% of cases can be explained by known chromosomal abnormalities [5]. Further, a recent study of twins suggests that genetic involvement may be overestimated in literature and that environmental factors may play a larger role than previously described [6]. Potential environmental risk factors include maternal diet [7], maternal hospital-associated bacterial infections during pregnancy [8], maternal exposure to air pollutants [9], parental age [4], and infant nutrition [10]. Recent studies have also shown relationships between maternal anti-cerebellar antibodies and the severity in autism in children [11].

Post-mortem analyses and neuroimaging studies of individuals with autism have provided insight into the pathology of the disease, including dysfunctional neuronal synapses, decreased cerebellar gray and white matter, enlargement of the amygdala, and abnormal growth patterns in the frontal cortex [4,12]. The mechanism by which these neurological effects take place is still unanswered; over the last decade, particular interest has emerged in studying the potential connection between levels of different fatty acids in body tissues and the occurrence of autism, as fatty acids play a crucial role in brain development [10,12,13,14]. Fatty acids make up 60% of the dry weight of the brain, and 20% are long-chain polyunsaturated fatty acids (PUFA) [14,15]. The two most abundant PUFA in the brain are omega-3 docosahexaenoic acid (C22:6 n-3; DHA) and omega-6 arachidonic acid (C20:4 n-6; AA). These PUFA play an important role in the composition of neuronal membranes, which in turn affects membrane fluidity and the structure and function of transmembrane proteins. One class of affected transmembrane proteins is the G-coupled protein receptors (GPCRs), which modulate propagation of signals at neuronal synapses and affect downstream signaling pathways and gene expression [16,17]. Concentration of DHA is high at neuronal synapses and has been shown to positively correlate with sodium potassium pump activity in the brain, demonstrating its importance in the maintenance of neuronal membrane potentials [13,16]. Phospholipids are a particularly relevant type of lipid to be studied in relation to autism, as they are highly abundant in the neuronal membranes and their fatty acid composition is known to reflect long-term fat intake and storage [18,19]. The red blood cell (RBC) phospholipid fatty acid profile is also thought to be a reliable biomarker for fatty acid status in tissues and organs, including the brain [20].

Small studies comparing the PUFA levels of plasma or RBC phospholipids between autistic and control individuals have reported mixed results. For example, Vancassel *et al.* [14] compared plasma phospholipid levels between 15 autistic children and 18 mentally delayed control participants. They found that total n-3 PUFA were significantly lower in the autistic test group, while AA and DHA levels were moderately reduced. Similarly, Bell *et al.* [18] found significantly lower AA levels in the RBC phospholipids of individuals with regressive autism (*n =* 18) than in controls (*n =* 55). A more recent study by Bell and colleagues [21] found that total n-6 PUFA were lower in the autistic test group (*n =* 45) than in pair-matched developmentally delayed controls. Conversely, Bu *et al.* [22] showed no significant differences in RBC membrane phospholipids of PUFA levels between autistic individuals and age-matched controls (*n =* 20). The largest study to date comparing plasma phospholipids of autistic (*n =* 153) and typically developing children (*n =* 97) was conducted by Weist and colleagues in 2009 [5]. They found that in the phospholipid class of phosphatidylcholine, DHA was significantly lower in the autistic group than in the general population, while phospholipid AA levels were not significantly different between the groups, although AA was found to be significantly lower in free fatty acids of the autistic participants.

While both plasma and RBC phospholipids have been found to correlate with the PUFA status of other tissues, PUFA levels in plasma and in RBC phospholipids have also been found to differ considerably from each other [19,23]. Notably, data produced by Bell *et al.* [21] indicates that weight percentages of phospholipid PUFA differed between plasma and RBC samples in autistic individuals. Since the largest fatty acid profiling study to date only analyzed plasma PUFA, a large study of the PUFA content of RBC phospholipids in autism is warranted.

One hypothesis regarding a potential mechanism for lower PUFA levels in autistic individuals is that the PUFA metabolism pathway may be overactive in autism, leading to rapid conversion from AA and DHA to their respective eicosanoids [24]. Altered lipid metabolism has been linked to other neurological disorders, including attention deficit disorder and schizophrenia [18,25,26]. Small studies with autism have also shown that children with autism have increased markers of lipid peroxidation [27], evidence of mitochondrial dysfunction [28], and increased levels of AA metabolites [29]. An increased level of the pro-inflammatory AA metabolite, prostaglandin E2 (PGE2), increases the risk of neuroinflammation, which can lead to excessive production of reactive oxygen species (ROS). High levels of ROS can cause DNA damage, proteolysis, and lipid damage, affecting the growth, development, and migration of neurons [30,31]. In a small study, Meguid *et al.* [30] found that autistic children had lower levels of the antioxidants glutathione peroxidase and superoxide dismutase, necessary to combat ROS damage. Importantly, DHA has been found to increase levels of the anti-oxidant glutathione [31]. Further, lipoxins derived from AA and resolvins and neuroprotectins derived from DHA help counteract neuroinflammation [16,31]. Rapid PUFA metabolism may therefore decrease levels of these anti-inflammatory molecules.

**Figure 1:**
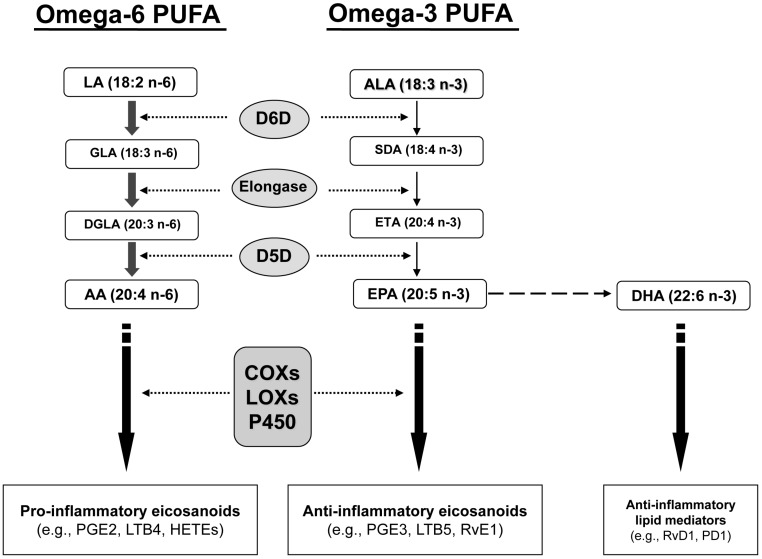
Diagram of the metabolic pathways for n-6 and n-3 polyunsaturated fatty acids (PUFA). Omega-6 linoleic acid (LA) and n-3 α-linolenic acid (ALA) cannot be synthesized by mammalian cells and must be obtained through the diet. They can be further elongated and desaturated by δ-6-desaturase (D6D), elongase, and D5D to form n-6 arachidonic acid (AA) and n-3 eicosapentaenoic acid (EPA), respectively. AA and EPA can then be converted by cyclooxygenases (COX), lipooxygenases (LOX), and cytochrome P450 (CYP 450) into pro- and anti-inflammatory eicosanoids, including prostaglandins (PG), leukotrienes (LT), thromboxanes (TX), hydroxyeicosatraenoic acids (HETE), and resolvins (Rv). The n-3 docosahexaenoic acid (DHA) can be converted into Rvs and protectins (PD).

As the pathway of PUFA metabolism in Figure 1 shows, metabolism of both n-6 and n-3 PUFA occurs through the same pathway of desaturase and elongase enzyme activity, which convert the essential substrate n-6 linoleic (LA) and n-3 α-linolenic (ALA) PUFA into longer-chain fatty acids, notably n-6 AA and n-3 EPA/DHA, respectively. Through the cyclooxygenase (COX) and lipoxygenase (LOX) pathways, AA and EPA are converted into eicosanoids and lipid mediators. Since PUFA are critically important in brain maturation, rapid conversion of these fatty acids to their metabolites may be implicated in the neurological developmental abnormalities present in individuals with autism.

The present study is the largest study to our knowledge to analyze the RBC phospholipid fatty acid profiles of autistic individuals (*n =* 121) and non-autistic, non-developmentally delayed controls (*n =* 110). We also tested the hypothesis that PUFA metabolism is more active in autistic individuals (*n =* 20) than in controls by measuring plasma levels of prostaglandin E2 (PGE2), a resulting eicosanoid of COX-mediated omega-6 PUFA metabolism.

## 2. Results and Discussion

### 2.1. Fatty Acid Profiling

**Figure 2:**
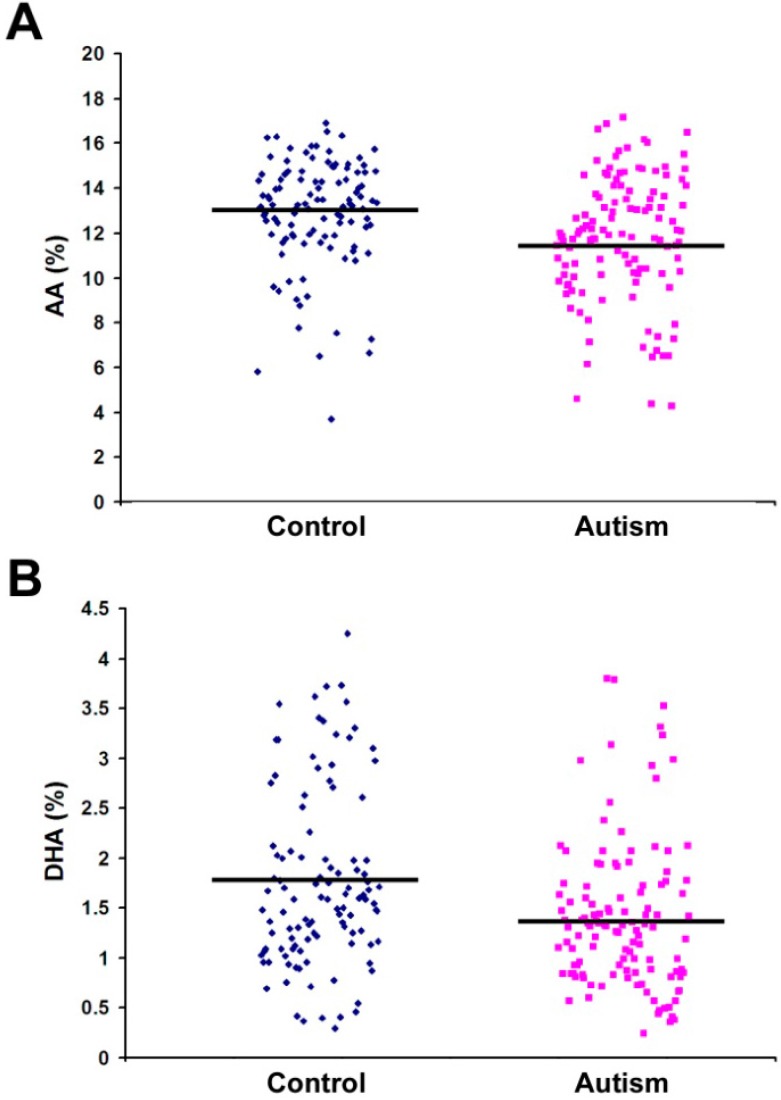

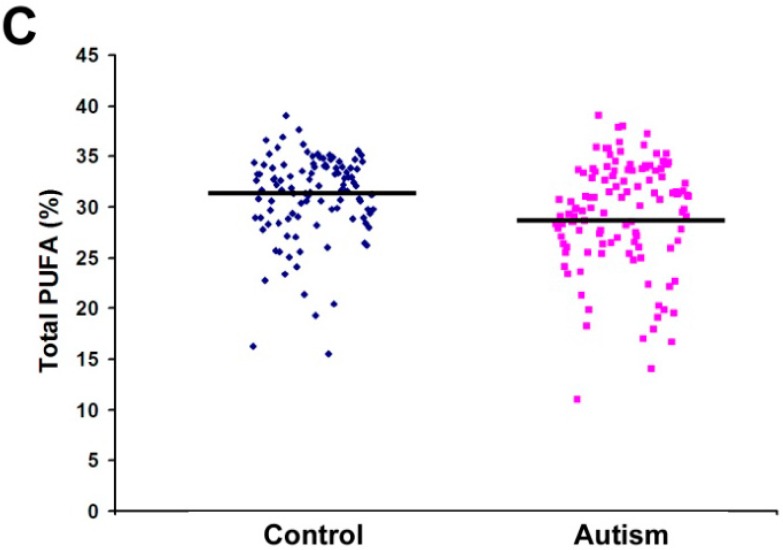
Scatterplot showing the distribution of fatty acid percentages of AA (**A**); docosahexaenoic acid (DHA) (**B**); and total PUFA (**C**) between control and autism groups. Phospholipids were extracted from red blood cell (RBC) samples of control subjects (*n* = 110) and autism patients (*n* = 121) and analyzed by gas chromatography. Bars represent the mean values (refer to Table 1 for significance).

To test the hypothesis that PUFA would be lower in the RBC membrane phospholipids of autistic individuals compared to controls, we conducted an independent-samples *t*-test assuming unequal variance to compare fatty acid compositions of the phospholipids of autistic individuals (*n =* 121) to those of non-autistic, non-developmentally delayed controls (*n =* 110). All subjects were aged 3–17 and most were Caucasian. The mean fatty acid percentages of all fatty acids tested are shown in Table 1. We found significant differences in several fatty acids between the autistic individuals and controls. Notably, a number of PUFA, mainly AA and DHA, were significantly lower in autistic individuals than in controls. The mean percentage of AA in autistic individuals was 11.736, compared to a control mean of 12.896 (*p =* 0.001). Similarly, the mean percentage of DHA in autistic individuals was 1.404, compared to a control mean of 1.757 (*p =* 0.001). The sum total of all tested n-6 and n-3 fatty acids were both significantly lower in autistic individuals compared to controls (Table 1). When AA and DHA data were re-analyzed after the removal of outliers (data more than two standard deviations from the mean), *p* values became even more significant (*p <* 0.00001). A scatterplot showing the distribution of AA, DHA, and total PUFA percentages is shown in Figure 2.

**Table 1:** Differences in fatty acid composition between autistic patients and unaffected individuals.

Fatty Acids	Autism (*n* = 121)		Control (*n* = 110)		*p*
	Mean	SD	Mean	SD	
**Saturates**					
C12:0	0.12	0.15	0.08	0.11	0.01 *
C14:0	0.66	0.40	0.57	0.29	0.06
C15:0	0.19	0.08	0.17	0.08	0.03 *
C16:0	27.63	3.14	26.37	2.47	0.001 *
C17:0	0.42	0.14	0.39	0.10	0.11
C18:0	19.63	2.23	19.66	2.18	0.93
C20:0	0.48	0.11	0.52	0.11	0.004 *
C22:0	1.28	0.42	1.30	0.32	0.58
C24:0	2.23	0.88	2.27	0.72	0.69
Total SFA	52.63	4.05	51.33	3.68	0.011 *
**Monounsaturates**					
C14:1	0.01	0.04	0.00	0.00	0.01 *
C16:1	0.39	0.38	0.36	0.30	0.44
C17:1	1.92	0.88	1.80	0.99	0.35
C18:1	13.35	2.88	12.96	2.11	0.25
C20:1	0.20	0.10	0.22	0.09	0.24
C22:1	0.22	0.24	0.17	0.12	0.04 *
C24:1	1.94	0.66	2.10	0.59	0.05
Total MUFA	18.03	5.18	17.61	4.21	0.23
**Polyunsaturates**					
C18:2 n-6	11.25	2.11	11.00	2.13	0.36
C18:3 n-6	0.01	0.03	0.01	0.02	0.3
C18:3 n-3	0.15	0.14	0.23	0.51	0.12
C20:2 n-6	0.20	0.11	0.22	0.09	0.37
C20:3 n-6	1.44	0.41	1.64	0.55	0.003 *
C20:4 n-6 (AA)	11.74	2.79	12.90	2.43	0.001 *
C20:3 n-3	0.01	0.06	0.00	0.01	0.36
C20:5 n-3	0.22	0.22	0.22	0.22	0.98
C22:2 n-6	0.01	0.03	0.02	0.06	0.04 *
C22:4 n-6	1.93	0.72	2.11	0.73	0.07
C22:5 n-3	0.97	0.39	0.97	0.39	0.97
C22:6 n-3 (DHA)	1.40	0.74	1.76	0.89	0.001 *
Total n-6	26.59	4.75	27.89	4.02	0.026 *
Total n-3	2.75	1.14	3.18	1.17	0.005 *
Total n-6:n-3	11.02	4.06	10.05	4.00	0.068
Total PUFA	29.34	5.31	31.06	4.21	0.007 *

* *p* < 0.05; Mean values are given as percentages of total fatty acid content measured; PUFA: polyunsaturated fatty acids.

### 2.2. Quantification of PGE2 Concentration

**Figure 3:**
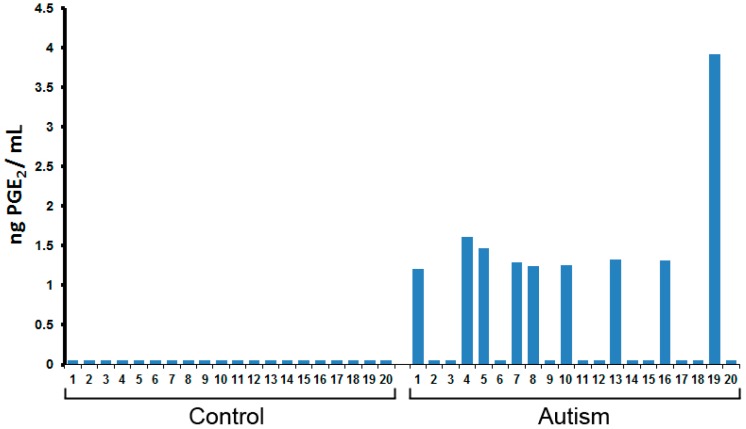
Plasma PGE_2_ levels of control subjects and autism patients. Plasma PGE_2_ levels were quantified by LC/MS for control subjects (*n* = 20) and autism patients (*n =* 20). All control samples were under the detection limit of <0.71 ng/mL. In contrast, PGE_2_ levels were detected in 9 of the 20 plasma samples from ASD individuals, ranging from 1.21 to 3.91 ng/mL.

PGE2 is a major metabolite of AA and is also a key inflammatory eicosanoid. To test our hypothesis that autistic individuals may exhibit higher levels of PGE2, we used LC/MS to quantify the plasma PGE2 levels for autistic individuals (*n =* 20) and controls (*n =* 20) (Figure 3). All control samples were under the detection limit for PGE2 detection of <0.71 ng/mL. In contrast, PGE2 levels were detected in 9 of the 20 plasma samples from autistic individuals, ranging from 1.21 to 3.91 ng/mL, indicating a marked difference in plasma PGE2 levels between autistic individuals and control subjects.

To our knowledge, the present study is the largest investigation of RBC phospholipids in autistic individuals. We demonstrated that levels of both n-6 and n-3 PUFA were significantly lower in autistic individuals than in non-autistic, non-developmentally delayed controls. We also found that plasma levels of the n-6 AA metabolite PGE2 were higher in autistic individuals than in controls. Because PUFA are integral for neuronal development and signaling, lower levels of these fatty acids and higher levels of a key n-6 metabolite suggest that fatty acid metabolism may be abnormal in individuals with autism.

Our findings of an altered phospholipid PUFA profile are consistent with previous findings of lower levels of total n-3 PUFA [14,18], n-6 AA [18], n-3 DHA [5], and total n-6 PUFA [21] in autistic individuals than in controls. Differences in total results among studies may result from different types of blood samples used, varying subsets of autism studied, and the type of control group recruited. For example, Vancassel and colleagues [14] analyzed plasma phospholipids and recruited mentally delayed individuals as the control group, while Weist *et al.* [5] compared plasma phospholipids between autistic individuals and the general population. Both Bell *et al.* [18] and the present study analyzed RBC phospholipids. The data by Bell and colleagues [21] provide interesting insight into the effect that different blood sampling types and control groups can have on the study outcome. This research team compared the differences in both erythrocyte phospholipids and plasma polar lipids between 45 autistic individuals and pair-matched typically developing controls. They also made a similar comparison using developmentally delayed (DD) individuals as the control group instead. They found that total n-6 PUFA were significantly lower only in the erythrocytes of the autistic group compared to DD controls. The essential PUFA precursor of AA, LA (C18:2 n-6), was significantly lower only in the erythrocytes of autistic patients compared to pair-matched typically developing controls. No PUFA were found to be significantly different when plasma samples were compared between groups. This data highlights the complexities of studying fatty acid profiles and the necessary caution that must be taken in making comparisons among studies.

A potential limitation of the present study is that we did not conduct an age-matched nor gender-matched analysis between the autistic and control groups. Gender-specific fatty acid differences in other studies demonstrate this limitation [5]. In addition, a study comparing RBC phospholipids of 88 healthy children aged 1–15 indicated that while most fatty acids stabilized after age two, ALA (C18:3 n-3) and trans fatty acids continued to increase throughout childhood [32].

In analyzing PUFA content, we specifically chose to analyze the fatty acid composition of RBC membrane phospholipids because they are known to reflect long-term lipid storage and therefore should not be affected by food intake directly prior to the participants’ blood drawing [19]. However, we cannot discount the possible effects of long-term fatty acid profile differences as a result of restricted food preferences of individuals with autism [33,34].

Another major finding of our study is the increase in plasma levels of PGE2 in autistic patients. We focused our analysis on the n-6 AA metabolite PGE2, rather than n-3 PUFA metabolites, due to the much higher levels of n-6 PUFA in RBC membranes than n-3 PUFA. Since PUFA levels have been found to positively correlate with levels of their metabolites [35,36], we expected n-6 PUFA metabolite levels to be easier to quantify. Our finding that plasma PGE2 levels are higher in autistic individuals than in controls is in agreement with a previous study by El-Ansary *et al.* [29]. As shown in Figure 1, COX and LOX enzymes convert n-6 and n-3 PUFA into pro- and anti-inflammatory eicosanoids, respectively. Because tissue levels of n-6 PUFA are much higher than levels of n-3 PUFA, rapid fatty acid metabolism would be expected to produce more pro-inflammatory eicosanoids, such as PGE2, than anti-inflammatory metabolites. Therefore, our findings that PUFA levels are lower in autistic individuals, and our findings that metabolite PGE2 levels were only detectable in the plasma of autistic individuals, support the hypothesis that lipid metabolism may be more active in autistic individuals than in controls. Increased levels of pro-inflammatory PGE2 in autistic patients may also be involved in increased neuroinflammation [31], which is thought to impair brain development and has been linked to autism in a number of studies [37,38,39]. Further existing evidence of abnormal lipid metabolism in autism includes the increased presence of the phospholipase enzyme responsible for breaking down phospholipids [18] and increased oxidative stress in autistic individuals compared to controls [27,30,40]. While lipid peroxidation levels are known to be a key marker of lipid degradation by oxidative stress, we were unable to measure lipid peroxidation directly due to varying levels of hemolysis in the plasma samples, which interfered with the colorimetric test for malondialdehyde, a marker for lipid peroxidation and oxidative stress.

Closer analysis is necessary to further investigate the potential presence of overactive lipid metabolism in autism. While we found PGE2 levels to be higher overall in autistic patients than controls, the number of samples tested was relatively small (*n =* 20 for each group). In addition, PGE2 has been recently measured in plasma [29], but PGE2 analysis may be more accurately performed using urine, due to the short half-life of prostaglandins in plasma [41]. We also cannot assume that our observations of both decreased PUFA levels and increased PGE2 levels in autistic individuals represent a cause-effect relationship, as PGE2 levels can be influenced by other factors such as diet [35,36]. For example, many children with autism suffer from gastrointestinal issues, including inflammation [42], which may involve increased production of pro-inflammatory prostaglandins such as PGE2 [43]. Larger studies are necessary to test multiple n-6 and n-3 markers of the PUFA metabolism pathway in order to better elucidate whether abnormal metabolism is responsible for the decreased PUFA levels and increased PGE2 levels in autistic individuals, or if dietary factors play a role in these findings.

Although we did not collect dietary information from the study participants, we do not expect dietary differences between the groups to be the primary explanation for the significantly lower PUFA levels in the RBC membranes and higher PGE2 levels in the plasma of autistic individuals. If diet were the primary cause of this result, then we would expect lower PGE2 levels in autistic individuals compared to controls, because levels of the AA metabolite, PGE2, have been shown to positively correlate with AA intake levels [35,36]. However, we found the opposite; autistic individuals had higher PGE2 levels in their plasma than controls, as we would expect if fatty acid metabolism were overactive in individuals with autism.

If overactive PUFA metabolism is indeed lowering PUFA levels in the tissues of autistic individuals, then supplementation with n-3 DHA may be a useful intervention as an alternative approach to the management of autism. DHA, one of the PUFA found in this study to be significantly lower in autistic individuals than controls, is integral for neuronal development and reduces tissue inflammation. Small clinical studies testing the effects of n-3 PUFA supplementation on individuals with autism have produced mixed results. Some small studies have demonstrated that n-3 PUFA supplementation may help ameliorate clinical symptoms of autism. For example, Yui *et al.* [44] found that combined AA and DHA supplementation improved social withdrawal and communication in individuals with autism compared to individuals who received a placebo. In addition, Meguid *et al.* [30] found that supplementation with a supplement high in DHA significantly decreased the CARS score in individuals with autism, indicating behavioral improvement after n-3 supplementation. A recent meta-analysis concluded that, to date, there is not enough quality evidence to support n-3 PUFA supplementation in the treatment of autism, citing sample size and control measures as weaknesses in existing studies [45]. Larger clinical studies are therefore necessary to determine whether or not n-3 supplementation improves the symptoms of ASD and reduces markers of neuroinflammation.

Our study also highlights the importance of fatty acids in brain structure and function associated with disease development. Jones *et al.* [46] recently reported that a maternal diet rich in n-6 PUFA and deficient in n-3 PUFA during gestation and lactation may produce autistic-like deficits in offspring, supporting the notion that PUFA are key factors in prenatal and early life brain development related to social behaviors.

In conclusion, autism is a highly complex disorder with many unanswered questions about its pathogenesis. PUFA metabolism is an important area of ASD research because PUFA are fatty acids necessary for brain development and PUFA metabolites modulate the inflammatory response in the brain. The present study demonstrates that key PUFA required for brain development are significantly lower in individuals with autism compared to individuals without ASD, potentially due to overactive metabolism of PUFA in individuals with autism.

## 3. Experimental Section

### 3.1. Collection and Transportation of Blood Samples

Whole blood samples were collected at Children’s Hospital Boston as part of the patients’ clinical diagnostic workup for autism. Diagnosis of autism was based on the Diagnostic and Statistical Manual for Mental Retardation (DSM-IV) and the Childhood Autism Rating Scale (CARS). Blood samples from children aged 3–17 that met the DSM-IV and CARS scores for autism were considered our autistic test group. Patients on the broader autism spectrum, including those with Asperger Syndrome and PDD-NOS, were not included in the study. The control group consisted of children aged 3–17 who went to Children’s Hospital Boston for genetic testing unrelated to developmental delay, such as hearing loss without overlapping features of autism. Test and control samples were not age nor gender-matched, although autism samples were collected at a male:female ratio of 4:1. Fasting was not a requirement prior to blood draws. All blood samples in this study were leftover samples from the clinical diagnostic work-ups. 121 whole blood samples from autistic patients and 110 control samples were frozen at −80 °C and transported to Massachusetts General Hospital (MGH) on dry ice for RBC phospholipid analysis.

For plasma PGE2 analysis, 40 fresh whole blood samples (20 from autistic group and 20 controls) were obtained to reduce hemolysis. The fresh whole blood samples were temporarily stored at 4 °C, and transported to MGH on wet ice. Upon arrival at MGH, plasma was separated from whole blood by centrifuging at 3000 rpm for 10 min at 4 °C; RBC and plasma were then stored separately at −80 °C for further analysis.

The basic research portion of the project, conducted at Massachusetts General Hospital, was exempt from IRB approval, as all samples were coded to exclude all patient information.

### 3.2. Lipid Analysis of RBC Membrane Phospholipids

#### 3.2.1. Lipid Extraction

Lipid extraction and methylation were performed as previously described [47]. Whole blood sample was thawed on ice. 200 μL was transferred to a 10 mL capped glass vial and centrifuged at 3000 rpm for 10 min at 4 °C to pellet the cells. Supernatant was removed and the pellet was washed with 1 mL of 0.9% saline solution. Centrifugation and supernatant removal were repeated once. 1 mL of miliQ water was added to the pellet to lyse the cells. The solution was vortexed and centrifuged again at 3000 rpm for 10 min at 4 °C. Supernatant was discarded. 5 mL of lipid extraction reagent (chloroform:methanol 2:1 plus 0.005% butylated hydroxytoluene) was added to the cell pellet; the vial was sealed under nitrogen and vortexed at 4 °C for 30 min. 5 mL of 0.9% saline solution was added to the vial. The solution was vortexed and centrifuged at 3000 rpm for 10 min at room temperature (RT) to separate aqueous and organic layers. The organic chloroform (bottom) layer was transferred to a glass culture tube and allowed to dry under a steady flow of nitrogen and reconstituted in 40 μL of lipid extraction reagent.

#### 3.2.2. Thin Layer Chromatography (TLC)

Phospholipid isolation was performed with TLC under the following conditions: before sample loading, the silica gel-coated glass TLC plate was dried at 80 °C for 1 h; development buffer makeup was 80 mL petroleum ether: 20 mL diethyl ether: 1 mL acetic acid. After development, the plate was sprayed with anilino naphthalene sulfonic acid (ANSA) coloring reagent and visualized under UV light. The phospholipid layer was extracted from the gel and transferred to a clean 10 mL glass vial.

#### 3.2.3. Fatty Acid Methylation

To the extracted phospholipid silica gel layer, 1.5 mL hexane and 1.5 mL 14% boron triflouride-methanol solution was added. The vial was sealed under nitrogen, capped tightly, and heated on a dry heat block at 100 °C for 1 h to convert the fatty acids of phospholipids to fatty acid methyl esters. Once cool, 1 mL of miliQ water was added to the vial; the vial was vortexed and centrifuged at 3000 rpm for 10 min at RT. The hexane layer containing the fatty acids was extracted, transferred to a glass culture tube, and dried under a steady stream of nitrogen gas. The dried fatty acid samples were then reconstituted in 50 μL of hexane for GC analysis.

#### 3.2.4. Gas Chromatography

Fatty acid profiles were determined using gas chromatography. The fatty acid methyl esters were injected onto a fully automated Agilent 6890N Network GC system, with a 7683 Series Injector, equipped with a flame ionization detector (Agilent Technologies, Santa Clara, CA, USA). The chromatography used an Omegawax 250 capillary column (30.0 m × 250 µm × 0.25 µm nominal) (Cat. No. 24136; Sigma-Aldrich, St. Louis, MO, USA). Samples were injected at a volume of 2.0 µL and a split ratio of 15:1. The total run time was 57 min, with the following conditions: initial oven temperature of 130 °C, hold for 3 min, ramp at 5 °C/min up to 180 °C, ramp at 2.5 °C/min up to 200 °C, hold for 15 min, ramp at 1 °C/min up to 210 °C, hold for 5 min, ramp at 5 °C/min up to 240 °C. Peaks of the resolved fatty acids were identified by comparison of retention time with a reference standard (Cat. No. GLC-461; Nu-chek-Prep, Inc., Elysian, MN, USA), and area percentage for all resolved peaks was analyzed by using a Perkin-Elmer M1 integrator (Boston, MA, USA).

#### 3.2.5. Statistical Analysis

An independent *t*-test assuming unequal variance was performed to compare RBC phospholipid content (measured as percentages of total quantified fatty acids) between autistic and non-autistic individuals. Statistical significance was set as *p* < 0.05. In order to ensure that outliers did not skew the data of the AA and DHA fatty acids, data points that fell more than 2 standard deviations away from the mean (four and nine outliers for AA and DHA, respectively) were removed and the *t*-test was repeated to confirm significance.

### 3.3. Quantification of PGE2 from Human Plasma Samples

#### 3.3.1. Extraction

An aliquot of 350 μL plasma sample were added to 2.2 mL water. 20 μL of internal standard (D4-PGE2, 5 μg/mL, in ethanol) and 0.45 mL MeOH were added into the solution, vortexed for 1 min, and let to stand on ice for 1 h. The solution was centrifuged at 3000 rpm for 15 min, supernatant was collected and adjusted to pH 3.0 with 0.2 N HCl. The sample solution was then loaded on C-18 SPE column (preconditioned with 2 mL water and 2 mL MeOH) and washed with 1 mL water. PGE_2_ was then eluted with 2 mL ethyl acetate, condensed to dryness, and re-dissolved with 100 μL ETOH for LC/MS analysis.

#### 3.3.2. LC/MS Condition

Mobile Phase: A: 0.01% HOAc-H_2_O, B: 0.01% HOAc-ACN; Gradient: 0–14 min, 32% B, 16–20 min, 95% B, 22–25 min, 32% B; Flow Rate: 0.8 mL/min; Injection: 20 μL; Electrospray ionization in negative mode; Full scan from *m*/*z* 50 to *m*/*z* 500; Target: *m*/*z* 351; Nebulizer Pressure: 15.0 psi; Dry Gas: 5.0 L/min; Dry Temperature: 325 °C; Compound Stability: 20%; Average: 50.

#### 3.3.3. Quantification of PGE_2_

The level of PGE2 was quantified using an internal standard curve (concentration of PGE2 *vs.* the peak area ratio of PGE2 to D4-PGE2).

#### 3.3.4. Reagent Information

Acetonitrile (HPLC Grade) purchased from VWR International (Radnor, PA, USA); Water (HPLC Grade) purchased from VWR International; PGE2 standard and Internal standard D4-PGE2 purchased from Cayman Chemical; High Performance C-18 Solid Phase Extraction cartridge purchased from Agilent Technology.
